# Associations between serum inflammatory mediators and chronic pain after laparoscopic inguinal hernia repair in elderly patients

**DOI:** 10.3389/fnhum.2026.1846116

**Published:** 2026-08-03

**Authors:** Dongyang Ma, Anqing Lu, Yuanhong Li, Bin Li, Wenzhang Lei, Yinghan Song

**Affiliations:** 1 Division of Gastrointestinal Surgery, Department of General Surgery, West China Hospital, Sichuan University, Chengdu, Sichuan, China; 2 Department of Central Transportation, West China Hospital, Sichuan University, Chengdu, Sichuan, China; 3 Department of Anesthesiology, West China Tianfu Hospital, Sichuan University, Chengdu, Sichuan, China; 4 Department of General Surgery, West China Airport Hospital, Sichuan University, Chengdu, Sichuan, China; 5 Department of Day Surgery Center, West China Hospital, Sichuan University, Chengdu, Sichuan, China

**Keywords:** chronic pain, inflammation, inguinal hernia, interleukin-6, laparoscopy, NLRP3

## Abstract

**Objective:**

This study aimed to investigate the associations between serum inflammatory mediators and chronic pain after laparoscopic inguinal hernia repair in elderly patients.

**Methods:**

From March 2022 to June 2023, 116 elderly patients (≥65 years old) diagnosed with inguinal hernia were enrolled in this study. All patients underwent total extraperitoneal repair (TEP) by laparoscopic surgery. Blood samples were obtained from each patient within the first postoperative day. Serum profiles of IL-1β, IL-6, IL-18, HMGB1, and NLRP3 were determined by ELISA. After that, a 6-month follow-up was performed, and pain was assessed using the Visual Analog Scale (VAS). Chronic pain was defined as inguinal pain that persisted for at least 6 months. On the basis of this definition, patients were divided into a chronic pain group (CP group, *n* = 11) and a non-chronic pain group (Non-CP group, *n* = 105). Logistic regression analysis was applied to investigate significant predictors of postoperative chronic pain, and the predictive performance of related indicators was verified using receiver operating characteristic (ROC) curves.

**Results:**

Univariate logistic analysis revealed that the presence of diabetes, hernia ring defect diameter, severe hernia ring adhesions, prolonged time to resume daily activities, and recurrence of hernia were significantly associated with postoperative chronic pain (all *p* < 0.05). Additionally, serum indicators, including elevated neutrophil counts and serum levels of IL-1β, IL-6, CRP, HMGB1, and NLRP3, were elevated in patients with postoperative chronic pain (all *p* < 0.05). Multivariate logistic regression analysis revealed that IL-6 (OR = 1.99, 95% CI 1.28–3.10; *p* = 0.002) and NLRP3 (OR = 1.39, 95% CI 1.14–1.69; *p* = 0.001) independently predict increased risk factors for chronic pain. The results of the ROC curve revealed that elevated IL-6 and NLRP3 levels had excellent predictive value for chronic pain, with AUCs of 0.975 (95% CI 0.949–1.000) and 0.907 (95% CI 0.852–0.961), respectively.

**Conclusion:**

Hernia ring defect diameter and early postoperative serum levels of IL-6 and NLRP3 are independent risk factors for chronic pain after TEP in elderly patients. IL-6 and NLRP3 are promising exploratory candidate biomarkers that warrant further validation.

## 1 Introduction

Inguinal hernia (IH) is among the most common surgical pathologies in elderly patients, and its incidence continues to increase with age (Wang et al., 2030). With age, the gradual loss of tissue strength and increased intra-abdominal pressure are the main reasons for the higher prevalence in elderly individuals (Wu et al., 2017). Among them, men have a 12.4 times greater risk of developing IH than women do. However, patients who are overweight or obese have a relatively lower risk of IH (de Goede et al., 2015). Hernia repair surgery is currently a commonly used clinical treatment. Clinically, this operation is performed via two approaches, namely, open surgery and laparoscopic surgery (Halpern et al., 2024). Accumulating evidence has shown that during the postoperative period of hernia repair, pulmonary, cardiovascular, and urinary system complications are more frequent in geriatric patients (Gianetta et al., 1997). Additionally, chronic postoperative inguinal pain (CPIP) is a significant complication affecting quality of life after hernia repair, with an incidence rate as high as 17.01% (Chu et al., 2024). Patients rely on painkillers and physical therapy to alleviate pain, and some require secondary surgery to replace the mesh (Sanders and Kingsnorth, 2012) or even undergo triple neurectomy of the iliohypogastric nerve, ilioinguinal nerve, and genitofemoral nerve, significantly increasing the physical and financial burden on patients (Beel and Berrevoet, 2022).

One of the core mechanisms of CPIP is direct injury, compression, or traction of nerves in the inguinal region during surgery (Loos et al., 2007). In recent years, local and systemic inflammatory responses triggered by surgical trauma have been shown to play a critical role in the chronicity of pain (Luo et al., 2016). As a central component of innate immunity, the NLRP3 inflammasome can be activated by damage-associated molecular patterns. This activation leads to the cleavage of pro-caspase-1 into its active form, promoting the maturation and release of interleukin (IL)-1β and IL-18, thereby initiating a cascade of inflammatory responses (Chen and Smith, 2023). In studies on chronic postoperative ischemic pain, the activation of microglia and astrocytes in the spinal dorsal horn, along with the overexpression of NLRP3, has been shown to be crucial for pain maintenance (Zhang et al., 2022). Additionally, as a pleiotropic proinflammatory cytokine, IL-6 not only directly participates in peripheral and central sensitization processes but also mediates persistent pain states by activating astrocytes and microglia. These proinflammatory factors act directly on neurons and glial cells, exacerbating central sensitization and serving as key molecules in the persistence and amplification of pain signals.

Laparoscopic total extraperitoneal repair (TEP) has advantages such as minimal invasiveness and a low risk of intraperitoneal complications, making it one of the most commonly used surgical techniques in clinical practice (Andresen and Rosenberg, 2024). However, significant acute inflammatory reactions still occur postoperatively. Studies have shown that 48 h after TEP surgery, circulating CRP levels and white blood cell counts are significantly elevated compared with baseline values (Liew et al., 2017), indicating that surgical trauma activates systemic inflammatory responses. Nevertheless, there is still insufficient evidence regarding whether early postoperative systemic inflammation levels are associated with the risk of CPIP.

On the basis of the aforementioned background, this study retrospectively included 116 elderly (≥65 years) IH patients who underwent TEP surgery. Circulating profiles of IL-6, IL-1β, IL-18, NLRP3, and HMGB1 were measured within 24 h post-operatively, and the occurrence of chronic pain was assessed through a 6-month follow-up. This study aimed to identify independent risk factors for chronic pain in elderly patients after TEP surgery, evaluate the predictive efficacy of early inflammatory markers for chronic pain, and screen for high-value biomarkers with potential clinical applications. This study is expected to provide new evidence for elucidating the immunological mechanisms underlying the chronicization of pain after hernia repair in elderly patients and to offer an objective basis for early identification and precise intervention in high-risk patients.

## 2 Methods

### 2.1 Study design and ethical approval

In this study, elderly (≥65 years) IH patients were enrolled between March 2022 and June 2023. All the patients underwent elective TEP at West China Hospital. This study strictly adhered to the principles of the Declaration of Helsinki and was approved by the Biomedical Ethics Review Committee of West China Hospital, Sichuan University (No.: 2023–0204). Written informed consent was obtained from all participants before they were included in this research.

### 2.2 Patient selection

The inclusion criteria were as follows: (1) aged ≥65 years; (2) diagnosed with primary unilateral IH (oblique or direct hernia) by clinical and ultrasound examination; (3) underwent TEP surgery for the first time; (4) completed peripheral blood sample collection within 24 h post-operatively; and (5) completed clinical data and agreed to complete 6 months of postoperative follow-up.

The exclusion criteria were as follows: (1) emergency surgery; (2) femoral hernia; (3) concurrent abdominal surgery; (4) preoperative chronic pain, neuropathic pain, or long-term use of analgesic medications; (5) comorbid active infection, autoimmune disease, or malignancy; (6) preoperative long-term use of glucocorticoids or immunosuppressants; and (7) severe postoperative complications (e.g., mesh infection, deep vein thrombosis, cardiovascular or cerebrovascular events) requiring secondary surgery.

### 2.3 Data collection and variable definitions

Preoperative baseline data, including demographic characteristics (age, sex, BMI), and comorbidities (diabetes, hypertension, constipation, etc.), were collected through interviews and medical records. Intraoperative hernia characteristics, including hernia type (direct or indirect), maximum diameter of the hernia ring defect (cm), hernia location (left or right), severity of hernia ring adhesion (classified as mild, moderate, or severe), and operative time (min, from skin incision to wound closure), were recorded. Postoperative recovery indicators included postoperative hospital stay and time to resume daily activities.

### 2.4 Serum sample collection and inflammatory marker testing

All patients had 5 mL of peripheral venous blood collected within 24 h after surgery. The blood samples were centrifuged to separate the serum, aliquoted, and stored in a −80 °C freezer for testing. Serum concentrations of NLRP3 (Abcam, ab274401), IL-1β (Abcam, ab214025), IL-6 (Abcam, ab178013), IL-18 (Abcam, ab243091), and high-mobility group box 1 (HMGB1, Abcam, ab18256) were measured using ELISA kits. Serum CRP levels were determined by immunoturbidimetry on an automated biochemical analyzer (Mindray, BS-1,000 M). WBC and neutrophil counts were obtained from routine postoperative blood tests. All tests were performed by researchers who were blinded to the patients’ clinical outcomes, and duplicate wells were used.

### 2.5 Outcome definition and follow-up

All patients underwent regular follow-up, with assessments conducted by dedicated research nurses at 1 day, 1 month, and 6 months post-operatively through outpatient visits or structured telephone interviews. The Visual Analogue Scale (VAS) was used to assess pain intensity. This scale ranges from 0 to 10 points, with 10 points indicating the highest level of pain (Manangi et al., 2014). Aggravating factors of pain, such as exercise, abdominal strain, and cough, were recorded. Two comprehensive assessment tools, the Surgical Outcomes Measurement System (SOMS) (Forester et al., 2022) and the Carolinas Comfort Scale (CCS) (Heniford et al., 2008), were administered postoperatively. The SOMS postoperative questionnaire is aggregated into the same seven subscales (pain impact, pain quality, fatigue, physical functioning, body image, and satisfaction). This study focused on pain-related components within these domains: VAS pain, pain impact, and pain quality. The CCS questionnaire is regarded as the most commonly used validated quality of life (QOL) instrument for hernia surgery. It has 23 questions specific to pain, movement limitations, and the sensation of mesh experienced after surgery. Each question is scored on a 5-point scale (0 represents “no symptoms” and 5 represents “disabling symptoms”).

Primary outcome: Chronic pain was considered mild but bothersome or worse on the basis of thresholds of either SOMS scores ≥3/4 or CCS scores ≥2 (Patel et al., 2017). This threshold for significant pain was considered to be clinically relevant, representing daily symptoms or worse, and was based on similar definitions from previous reports (Forester et al., 2022; Patel et al., 2017).

### 2.6 Data analysis

SPSS Statistics 27.0 (Chicago, Illinois, United States) was used for data analysis. Continuous variables that are normally distributed are shown as the mean ± standard deviation. Two-sided independent Student’s *t* tests were performed for data difference analysis. The differences in nonnormally distributed variables, which are expressed as medians (interquartile ranges), were compared via the Mann–Whitney U test. Categorical variables were compared using chi-square tests or Fisher’s exact tests. To identify factors associated with CPIP, univariate logistic regression analysis was first performed. Variables with *p* < 0.10 in the univariate analysis and those with clear clinical significance were included in the multivariate analysis. The results are presented as odds ratios (ORs) and 95% confidence intervals (CIs). Given the limited number of chronic pain events (*n* = 11), we performed Firth’s penalized logistic regression to correct for rare-event bias. ROC curves were used to evaluate the predictive performance of various inflammatory markers for chronic pain at 6 months post-operatively, and the area under the curve (AUC) was calculated. The optimal cutoff values for predicting recurrence for each marker were determined by maximizing the Youden index (sensitivity + specificity − 1), and the corresponding sensitivity, specificity, positive predictive value, and negative predictive value were reported. Spearman’s rank correlation analysis was used to assess the correlation between the levels of inflammatory markers and the visual analog scale (VAS) score at 6 months post-surgery, and the correlation coefficient (r_s_) and its 95% CI (via Fisher Z transformation) were calculated, along with the corresponding *p* values. All the statistical tests were two-sided, and *p* < 0.05 was considered to indicate statistical significance.

## 3 Results

### 3.1 Baseline characteristics of the enrolled participants

**Figure 1:**
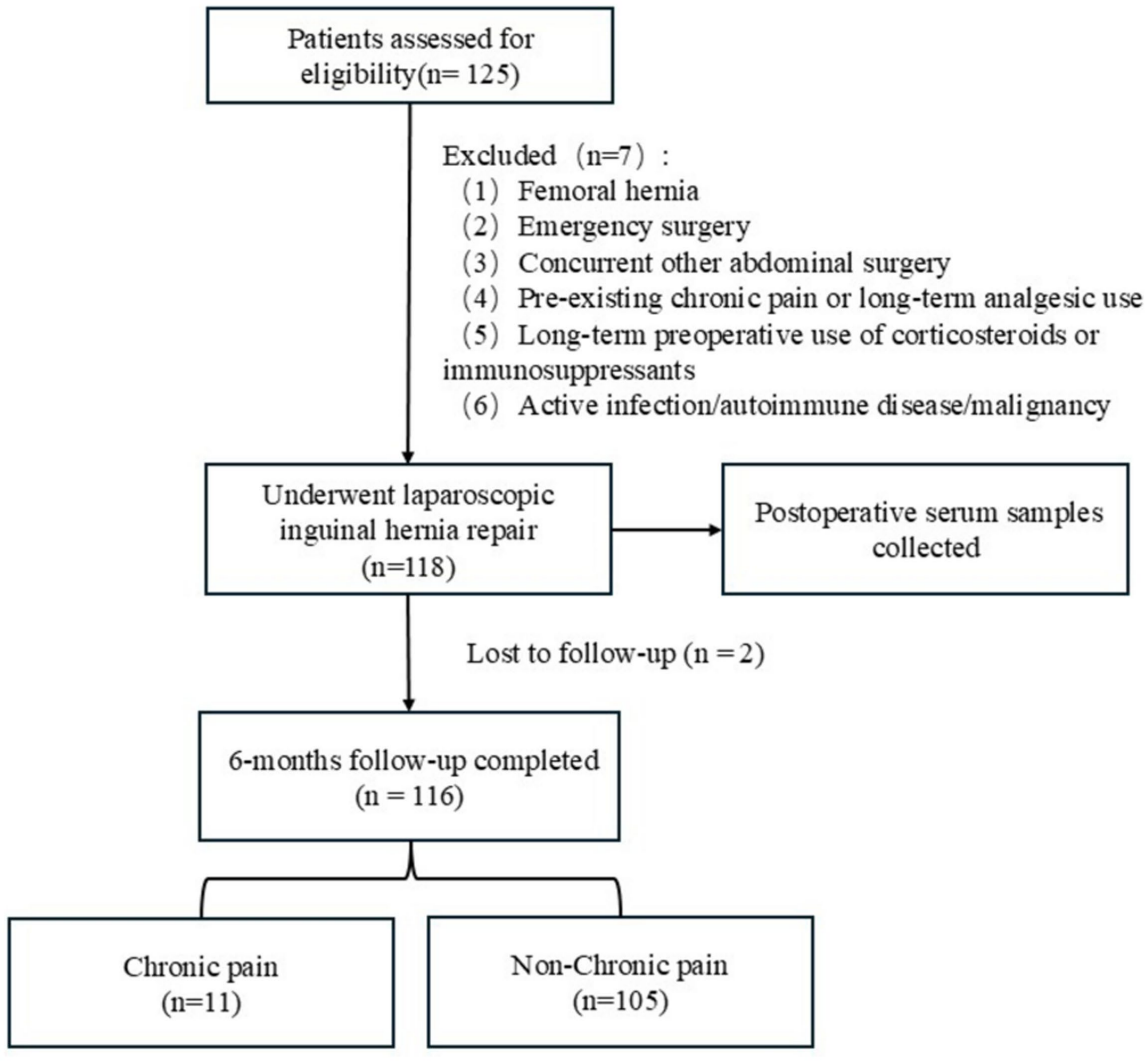
Flowchart of patient selection. A total of 125 patients with primary unilateral inguinal hernia who were scheduled for elective TEP were initially evaluated. After the exclusion of five ineligible patients, 118 patients underwent surgery, and postoperative serum samples were collected within 24 h of surgery. Two patients who completed 6 months of follow-up were lost to follow-up, leaving 116 patients included in the final analysis. Among them, 11 patients (9.5%) developed chronic pain (CP) within 6 months after surgery, while 105 patients (90.5%) had no chronic pain. TEP, totally extraperitoneal repair.

During the study period, a total of 125 eligible patients were screened. In terms of the exclusion criteria, 9 patients were not included (Figure 1). On the basis of the VAS scores 6 months after surgery, the 116 participants were divided into a chronic pain group (*n* = 11) and a nonchronic pain group (*n* = 105) (Table 1). The clinical parameters, including age, sex, body mass index (BMI), smoking history, hypertension status, hernia type, lesion side, and operation time, did not significantly differ between the two groups. However, the proportion of patients with diabetes was significantly greater in the chronic pain group than in the nonchronic pain group (72.7% vs. 36.2%, *p* = 0.042), and the degree of hernia ring adhesion was greater (*p* = 0.042). Compared with the nonchronic pain group, the chronic pain group had larger hernias (*p* < 0.001) and an increased proportion of hernias > 6 cm (*p* < 0.001). In terms of postoperative outcomes, the chronic pain group had a longer time to resume daily activities (*p* < 0.001) and a significantly greater recurrence rate (45.5% vs. 5.7%, *p* < 0.001).

**Table 1:** Baseline characteristics and postoperative inflammatory markers of patients in the chronic pain group and the nonchronic pain group.

Characteristic	Total (*n* = 116)	Chronic pain (*n* = 11)	Non-chronic pain (*n* = 105)	*P*-value
Age (years), median (IQR)	73.50 (8)	76.00 (11)	73.00 (8)	0.587
Male, n (%)	104 (89.7)	9 (81.8)	95 (90.5)	0.370
BMI (kg/m^2^), mean ± SD	25.70 ± 3.74	25.82 ± 3.05	25.68 ± 3.42	0.896
Smoke, n (%)	64 (55.2)	7 (63.6)	57 (54.3)	0.553
Diabetes, n (%)	46 (39.7)	8 (72.7)	38 (36.2)	0.042
Hypertension, n (%)	39 (33.6)	6 (54.5)	33 (31.4)	0.227
Constipation, n (%)	51 (39.5)	11 (61.1)	40 (36.0)	
Hernia size (cm), median (IQR)	4.94 (2)	6.80 (2)	4.78 (2)	<0.001
Hernia size category, n (%)				<0.001
<3 cm	11 (9.5)	1 (9.1)	10 (9.5)	
3–6 cm	81 (69.8)	2 (18.2)	79 (75.2)	
>6 cm	24 (20.7)	8 (72.7)	16 (15.2)	
Hernia type (oblique), n (%)	74 (63.8)	6 (54.5)	68 (64.8)	0.733
Location (Right side), n (%)	85 (73.3)	8 (72.7)	77 (73.3)	0.966
Degree of hernia ring adhesion, n (%)				0.042
mild to moderate	70 (60.3)	3 (27.3)	67 (63.8)	
severe	46 (39.7)	8 (72.7)	38 (36.2)	
Operation time (min), median(IQR)	36 (12)	36 (15)	36 (12.5)	0.832
Return to daily activities (days), median(IQR)	7 (3)	11(3)	7 (3)	<0.001
Recurrence	11 (9.5)	5 (45.5)	6 (5.7)	<0.001

Continuous variables with a normal distribution are presented as the mean ± SD and were compared using the independent samples *t*-test; those with a nonnormal distribution are presented as the median (IQR) and were compared using the Mann–Whitney U test. Categorical variables are presented as frequencies (percentages, %) and were compared using the chi-square test or Fisher’s exact test, as appropriate.

### 3.2 Differences in postoperative serum indicator levels

**Figure 2:**
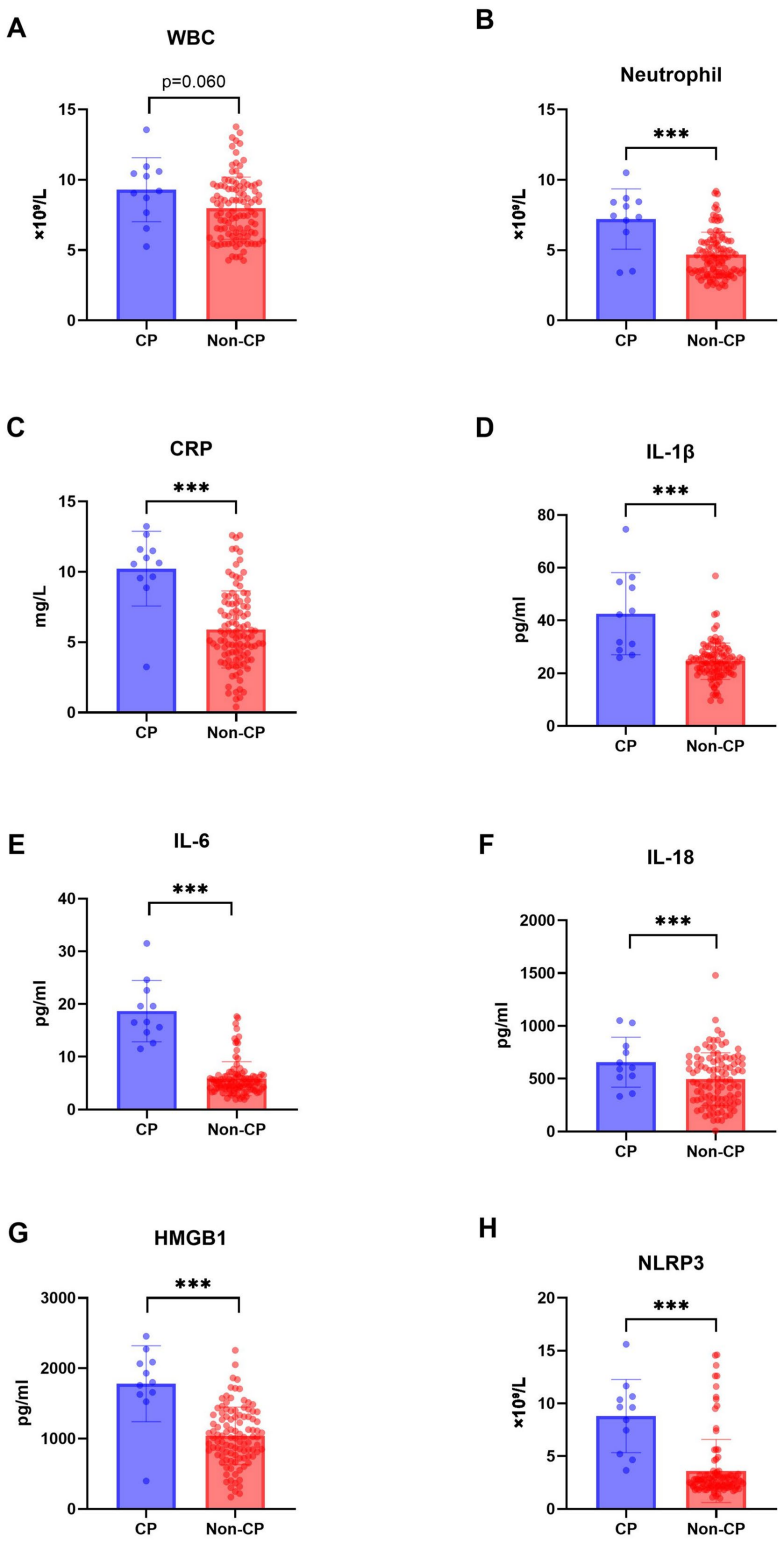
Comparison of postoperative inflammatory markers between the chronic pain group and the nonchronic pain group. Serum levels of **(A)** WBCs, **(B)** neutrophils, **(C)** CRP, **(D)** IL-1β, **(E)** IL-6, **(F)** IL-18, **(G)** HMGB1, and **(H)** NLRP3 were measured within 24 h after surgery. Dots represent individual data points. Patients in the chronic pain group (CP, *n* = 11) had significantly higher levels of neutrophils, CRP, IL-1β, IL-6, IL-18, HMGB1, and NLRP3 than those in the nonchronic pain group (non-CP, *n* = 105). No significant difference in WBC count was observed between the groups. Comparisons were performed using independent samples *t*-tests (for normally distributed data) or Mann–Whitney U tests (for nonnormally distributed data). *p < 0.05, **p < 0.01, ***p < 0.001.

We collected blood within 24 h after surgery and used ELISA to determine serum levels of CRP, IL-1β, IL-18, IL-6, HMGB1, and NLRP3. Serum CRP, IL-1β, IL-18, IL-6, HMGB1, and NLRP3 levels were significantly greater in the chronic pain group than in the nonchronic pain group (Figure 2). The neutrophil counts and serum profiles of IL-1β, IL-6, IL-18, HMGB1, and NLRP3 in the chronic pain group were significantly greater than those in the nonchronic pain group (all *p* < 0.05).

### 3.3 Correlations between inflammatory indicators and pain at 6 months post-surgery

**Figure 3:**
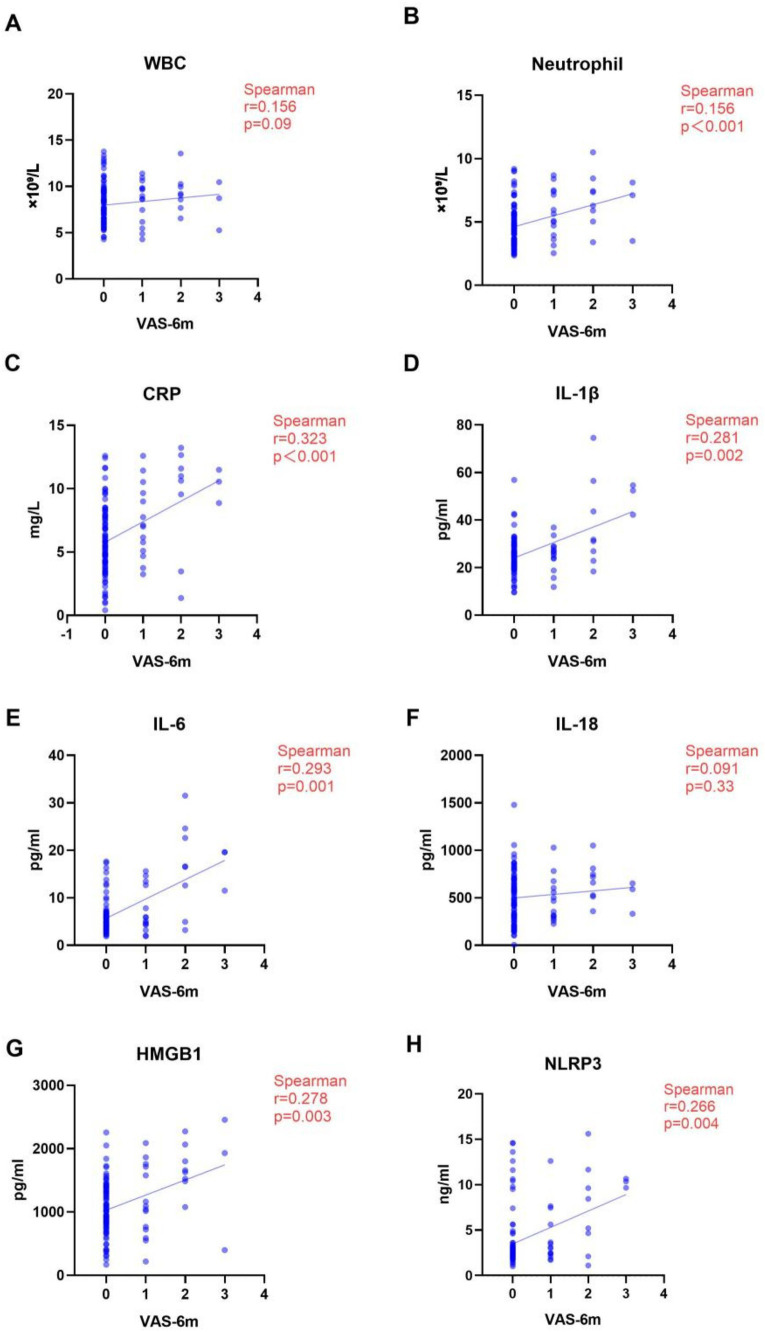
Correlations between inflammatory marker levels and VAS scores at 6 months post-surgery. Relationships between serum levels of **(A)** WBCs, **(B)** neutrophils, **(C)** CRP, **(D)** IL-1β, **(E)** IL-6, **(F)** IL-18, **(G)** HMGB1, and **(H)** NLRP3 measured within 24 h after surgery and VAS scores at 6 months post-surgery. Each dot represents an individual patient (*n* = 116). Spearman correlation coefficients (r) and *p*-values are shown in the upper right corner of each subplot. VAS, visual analog scale.

Spearman’s rank correlation analysis was conducted. The associations between serum inflammatory indicators and VAS scores were assessed (*n* = 116). CRP (*r* = 0.323, *p* < 0.001), IL-6 (*r* = 0.293, *p* = 0.001), IL-1β (*r* = 0.281, *p* = 0.002), HMGB1 (*r* = 0.278, *p* = 0.003), and NLRP3 (*r* = 0.266, *p* = 0.004) were significantly correlated with the VAS score at 6 months after surgery (Figure 3). The neutrophil count was also significantly correlated (*r* = 0.156; *p* < 0.001), whereas the white blood cell (*p* = 0.09) and IL-18 (*p* = 0.33) counts were not significantly correlated with the VAS score (Figure 3).

### 3.4 Univariate and multivariate logistic regression analyses

Univariate logistic regression analysis was conducted. Clinical parameters significantly associated with chronic pain included diabetes (OR = 4.70, 95% CI 1.18–18.79; *p* = 0.029), constipation (OR = 4.18, 95% CI 1.14–15.30; *p* = 0.031), maximum diameter of the hernia ring (OR = 2.70, 95% CI 1.52–4.81; *p* < 0.001), and time to resume daily activities (OR = 2.18, 95% CI 1.44–3.30; *p* < 0.001). Age, sex, BMI, smoking status, hypertension status, hernia type, lesion side, and operation time were not strongly associated with chronic pain (all *p* > 0.05) (Table 2). Among inflammatory indicators, WBC, CRP, IL-1β, IL-6, IL-18, HMGB1, and NLRP3 were significantly associated with chronic pain.

**Table 2:** Univariate logistic regression analysis of risk factors for chronic pain.

Parameters	ß	SE of ß	p Value	OR	95% CI
Age	0.042	0.057	0.462	1.043	0.932–1.167
BMI	0.012	0.094	0.895	1.012	0.842–1.218
Gender	0.223	0.8	0.78	1.25	0.261–5.993
Smoke	0.388	0.657	0.555	1.474	0.407–5.338
Diabetes	1.548	0.707	0.029	4.702	1.177–18.789
Hypertension	0.962	0.641	0.133	2.618	0.745–9.196
Constipation	1.43	0.662	0.031	4.177	1.141–15.299
Hernia size (cm)	0.993	0.295	<0.001	2.701	1.516–4.812
Hernia size category					
<3 cm vs. > 6 cm	−1.609	1.135	0.156	0.2	0.022–1.849
3–6 cm vs. > 6 cm	−2.983	0.837	<0.001	0.051	0.010–0.261
Hernia type (oblique)	−0.426	0.639	0.505	0.653	0.187–2.285
Location (left side)	0.031	0.712	0.966	1.031	0.255–4.164
Degree of hernia ring adhesion	−1.548	0.707	0.029	0.213	0.053–0.850
Operation time (min)	−0.006	0.04	0.876	0.994	0.918–1.075
Return to daily activities	0.777	0.212	<0.001	2.176	1.436–3.296
Recurrence	2.621	0.737	<0.001	13.75	3.242–58.316
WBC	0.252	0.14	0.071	1.287	0.979–1.692
Neutrophil	0.725	0.197	<0.001	2.066	1.404–3.040
CRP	0.558	0.149	<0.001	1.747	1.305–2.340
IL-1β	0.157	0.04	<0.001	1.17	1.082–1.267
IL-6	0.51	0.135	<0.001	1.664	1.278–2.167
IL-18	0.002	0.001	0.054	1.002	1.000–1.005
HMGB1	0.004	0.001	<0.001	1.004	1.002–1.006
NLRP3	0.311	0.079	<0.001	1.363	1.168–1.593

Given the limited number of chronic pain events (*n* = 11), two multivariate models were constructed with only two core predictors each to avoid overfitting (Table 3). Model 1 included hernia size and IL-6; both were independent risk factors for chronic pain (hazard size: OR = 3.47, 95% CI 1.35–14.06, *p* = 0.026; IL-6: OR = 1.87, 95% CI 1.40–3.30, *p* = 0.002). Model 2 included hernia size and NLRP3; both were also independent risk factors (hazard size: OR = 2.69, 95% CI 1.53–5.46, *p* = 0.002; NLRP3: OR = 1.40, 95% CI 1.18–1.74, *p* < 0.001). To validate the robustness of these findings, we performed Firth’s penalized logistic regression to correct for rare-event bias (Table 3). The results remained consistent: IL-6 (OR = 1.63, 95% CI 1.31–2.48; *p* < 0.001) and NLRP3 (OR = 1.37, 95% CI 1.16–1.67; *p* < 0.001) remained independent risk factors. To further validate the stability of the multivariate predictive models incorporating IL-6 and NLRP3, we performed bootstrap internal validation and Hosmer–Lemeshow calibration tests (Supplementary Table 1). The optimism-corrected AUC values were 0.959 for IL-6 and 0.890 for NLRP3. The Hosmer–Lemeshow tests demonstrated good calibration for both models (Supplementary Table 1).

**Table 3:** Multivariate logistic regression analysis of risk factors for chronic pain.

Models	Parameters	Standard multivariate analysis		Firth’s penalized regression	
		OR (95% CI)	*P*-value	OR (95% CI)	*P*-value
Model 1	Hernia size (cm)	3.47 (1.35–14.06)	0.026	2.69 (1.23–8.04)	0.013
	IL-6	1.87 (1.40–3.30)	0.002	1.63 (1.31–2.48)	<0.001
Model 2	Hernia size (cm)	2.69 (1.53–5.46)	0.002	2.45 (1.44–4.68)	<0.001
	NLRP3	1.40 (1.18–1.74)	<0.001	1.37 (1.16–1.67)	<0.001

Firth’s penalized regression was used to correct for rare-event bias (only 11 CPIP cases). Each multivariate model was limited to two core predictors to avoid overfitting. Bold values indicate statistical significance (*P* < 0.05). OR, odds ratio; CI, confidence interval.

### 3.5 The predictive value of serum markers for chronic pain

**Figure 4:**
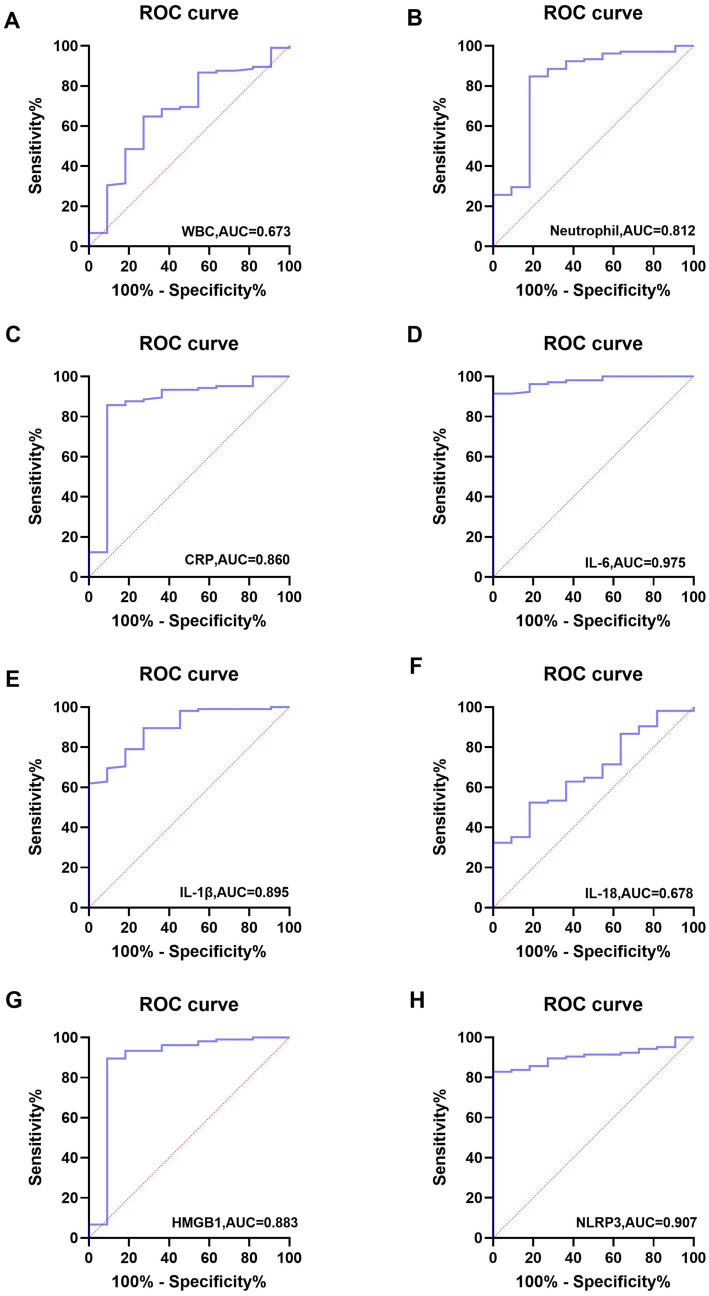
Receiver operating characteristic curves of postoperative inflammatory markers for predicting chronic pain at 6 months. ROC curves for **(A)** WBCs, **(B)** neutrophils, **(C)** CRP levels, **(D)** IL-1β levels, **(E)** IL-6 levels, **(F)** IL-18 levels, **(G)** HMGB1 levels, and **(H)** NLRP3 levels. All markers were measured within 24 h after surgery. The diagonal dashed line represents the reference line (AUC = 0.5). IL-6 exhibited the highest predictive efficacy (AUC = 0.975), followed by NLRP3 (AUC = 0.907). AUC: area under the curve; ROC: receiver operating characteristic; WBC, white blood cell; CRP, C-reactive protein; IL-1β: interleukin-1β; IL-6: interleukin-6; IL-18: interleukin-18; HMGB1: high mobility group box 1; NLRP3: NLR family pyrin domain containing 3.

The results of the ROC curve analysis revealed that various inflammatory markers have certain predictive value for chronic pain at 6 months after surgery (Table 4; Figure 4). Among them, IL-6 demonstrated the highest predictive efficacy (AUC = 0.975, 95% CI 0.949–1.000; *p* < 0.001), with an optimal cutoff value of 11.38 pg./mL, corresponding to a sensitivity of 100.0%, a specificity of 8.6%, and a Youden index of 0.914. NLRP3 showed the next highest predictive efficacy, with an AUC of 0.907 (95% CI 0.852–0.961; *p* < 0.001), an optimal cutoff value of 3.625 ng/mL, a sensitivity of 100.0%, a specificity of 17.1%, and a Youden index of 0.829. The area under the curve (AUC) values for the other inflammatory factors were as follows: 0.895 (95% CI 0.813–0.978), 0.883 (95% CI 0.728–1.000), 0.860 (95% CI 0.716–1.000), and 0.812 (95% CI 0.662–0.978). The predictive values of IL-18 and WBC were relatively low, with AUCs of 0.678 and 0.673, respectively.

**Table 4:** Predictive performance of postoperative inflammatory markers for chronic pain.

Characteristic	AUC (95% CI)	Cut-off (95% CI)	Sensitivity (95% CI)	Specificity (95% CI)	Youden index	*P*-value
WBC (×10^9^/L)	0.673 (0.504–0.842)	8.72 (6.54–10.60)	0.727 (0.464–0.990)	0.648 (0.556–0.739)	0.375	0.060
Neutrophil (×10^9^/L)	0.812 (0.662–0.978)	6.30 (6.30–8.12)	0.818 (0.590–1.000)	0.848 (0.779–0.916)	0.666	<0.001
CRP (mg/L)	0.860 (0.716–1.000)	8.87 (8.87–10.55)	0.909 (0.739–1.000)	0.857 (0.790–0.924)	0.766	<0.001
IL-1β (pg/mL)	0.895 (0.813–0.978)	31.13 (25.92–43.60)	0.727 (0.464–0.990)	0.895 (0.837–0.954)	0.619	<0.001
IL-6 (pg/mL)	0.975 (0.949–1.000)	11.50 (11.50–15.60)	1.000 (1.000–1.000)	0.914 (0.861–0.968)	0.914	<0.001
IL-18 (pg/mL)	0.678 (0.533–0.823)	513.80 (332.90–808.60)	0.818 (0.590–1.000)	0.524 (0.428–0.619)	0.342	<0.001
HMGB1 (pg/mL)	0.883 (0.728–1.000)	1526.50 (1526.50–1758.30)	0.909 (0.739–1.000)	0.895 (0.837–0.954)	0.804	<0.001
NLRP3 (ng/mL)	0.907 (0.852–0.961)	3.65 (3.65–7.46)	1.000 (1.000–1.000)	0.829 (0.756–0.901)	0.829	<0.001

AUC, area under the curve; CI, confidence interval. The cutoff values were determined by maximizing Youden’s index. All inflammatory markers were measured within 24 h after surgery.

## 4 Discussion

Investigating novel predictive factors of chronic pain following TEP is highly important for developing effective therapies. In this study, we reported that the incidence of chronic pain after TEP in elderly patients was 9.5%. We further evaluated serum inflammatory mediator levels in elderly patients following TEP. Multiple analyses revealed that early postoperative serum IL-6 and NLRP3 levels were potential independent risk factors for chronic pain in this cohort, suggesting their value as exploratory candidate biomarkers that warrant further validation.

CPIP is a common complication after hernia repair and has a negative effect on the quality of life of patients. A meta-analysis including 18 studies revealed that the pooled incidence of CPIP was as high as 17.01% (Chu et al., 2024). Another Portuguese multicenter cohort study reported an overall incidence of 16.6% (Cancer Genome Center, 2024). In this study, the incidence of CPIP in elderly patients was 9.5%, which was slightly lower than the levels reported in the literature. This may be related to the patient population and intervention methods used in this study. First, all the patients in this study were older than 65 years and had decreased pain sensitivity (Dubský et al., 2022). A retrospective study indicated that the CPIP group consisted of younger patients (54.4 years vs. 60.3 years), and younger age was considered a risk factor for CPIP (Widder et al., 2023). Second, all patients in this study underwent elective TEP surgery. Currently, laparoscopic techniques (including TAPP and TEP) have gradually become the primary treatment for IH (Andresen and Rosenberg, 2024). Compared with open surgery, laparoscopic surgery offers advantages such as minimal trauma, shorter hospital stays, and lower postoperative infection rates, making it an important treatment modality for elderly IHs (Al-Sharabi et al., 2025). Numerous studies have demonstrated that TEP surgery significantly reduces postoperative pain in IH patients (Haque et al., 2022; Ulutas and Yilmaz, 2025). Additionally, differing definitions of chronic pain can lead to variations in reported disease incidence. In this study, 18 patients had a VAS score ≥1 at 6 months post-operatively, but only 11 patients (9.5%) met the criteria for chronic pain. Future clinical practices and research need to establish clear operational definitions of chronic pain to avoid overestimating its incidence. Furthermore, previous studies have suggested that right-sided IH and indirect IH are most common in the elderly population (Hernandez-Rosa et al., 2011; Karatas et al., 2023). In our study, the higher proportion of indirect and right-sided hernias among patients reflects clinical representativeness.

The core finding of this study is that early postoperative serum IL-6 and NLRP3 levels have independent predictive value for chronic pain in elderly patients after TEP surgery. Chronic pain has long imposed significant pressure on healthcare systems worldwide, and its molecular mechanisms involve inflammatory molecules that activate nociceptors in the peripheral nervous system. These mechanisms are associated with inflammation and play a critical role in maintaining pain signal transmission (McMasters and Mora, 2025). The NLRP3 pathway is triggered by a variety of host “danger” situations, especially during infection. Metabolic dysregulation, which is accompanied by the release of “dangerous molecules,” can also lead to NLRP3 pathway activation and the subsequent release of IL-1β and IL-18 (Chen and Smith, 2023). In addition, as a pleiotropic proinflammatory cytokine, IL-6 is involved in the pathophysiological process of a variety of inflammatory diseases. However, in chronic inflammation, IL-6 and NLRP3 are not independent pathways and are activated simultaneously in many inflammatory diseases, such as rheumatoid arthritis (Wang et al., 2020). IL-6 can activate the NLRP3 inflammasome through direct action and transcriptional regulation, whereas the activated NLRP3 inflammasome drives the massive production of IL-6 through the release of factors such as IL-1β (Sonowal et al., 2022; Wang et al., 2020). This forms a positive feedback loop that amplifies inflammatory responses and promotes the persistence of pain.

The transmission of peripheral inflammatory signals to the center is a key step in the chronicity of pain. Peripheral surgical trauma activates local immune cells and the release of proinflammatory cytokines such as IL-6 and Il-1β. On the one hand, these factors directly sensitize peripheral nociceptors, and on the other hand, they can enter the central nervous system through the blood–brain barrier (Mokhtari et al., 2024). Once centrally located, these peripheral signals activate microglia and astrocytes in the spinal cord and brain. Activated microglia further release a large number of proinflammatory mediators and disturb the excitation–inhibition balance of the central nervous network by enhancing glutamatergic synaptic transmission and weakening GABAergic inhibitory signals, thereby inducing and maintaining central sensitization (Mokhtari et al., 2024). Recent studies have shown that NLRP3 plays important roles in the maintenance of neuropathic pain and chronic postoperative pain and is expected to become a new therapeutic target (Hu et al., 2022; Kui et al., 2025; Meng et al., 2021). Specifically, extracellular IL-1β and IL-18 directly act on neurons to reduce their excitation threshold, leading to central sensitization and hyperalgesia (Chen and Smith, 2023). IL-6 also plays a key role in this process. Basic studies have shown that IL-6 not only directly activates peripheral nociceptors but also acts on the central nervous system through the blood–brain barrier, activates astrocytes and microglia, and further enhances the excitability of spinal dorsal horn neurons through the JAK/STAT3 signaling pathway. Together with the NLRP3/IL-1β pathway, it promotes the establishment and maintenance of central sensitization and induces central sensitization (Lee et al., 2023). IL-6 and the NLRP3/IL-1β pathway jointly promote the establishment and maintenance of central sensitization and promote the transformation of acute pain to a chronic state.

The clinical consistency of the findings in this study is supported by those of previous studies. Clinical studies have confirmed that perioperative IL-6 levels are associated with the risk of chronic pain following various surgeries (e.g., arthroscopy surgery and thoracic surgery), and consistent suppression of its expression can significantly alleviate postoperative pain (Chu et al., 2026; Zhong et al., 2024). This study extends these findings to elderly patients undergoing IH repair surgery and is the first to validate the predictive value of IL-6 in patients after TEP surgery. Clinical studies have indicated that the activation level of the NLRP3 inflammasome in peripheral blood is significantly elevated in patients with neuropathic pain and is positively correlated with pain intensity (Wang et al., 2024). This study also revealed that NLRP3 is not only independently associated with chronic postoperative pain but also has high predictive efficacy (AUC = 0.907). Future studies should further validate these findings in large-sample, multicenter cohorts and explore whether blocking the key nodes of this circuit can effectively prevent or alleviate chronic postoperative pain.

This study has certain limitations. First, the sample size was relatively small, with only 11 patients in the chronic pain group, which affected the stability of the multivariate analysis and subgroup analysis. We avoided multicollinearity by constructing two separate multivariate models (including those for IL-6 and NLRP3), but studies with larger sample sizes are still needed for further confirmation. Second, inflammatory markers were detected only at a single time point (24 h after surgery), which does not reflect the dynamic trajectory of the inflammatory response. The peak times of different cytokines are different, and the cumulative inflammatory burden over time may be more valuable than a single measurement. Future research should consider conducting serial tests (such as before surgery and 6, 12, 24, 48, and 72 h after surgery) to construct the trajectory of inflammatory changes and determine the optimal timing for biomarker detection. Third, this was a single-center study conducted in a tertiary hospital in China; thus, our results may not be generalizable to other populations and medical environments. All patients were elderly (≥65 years old) and underwent only TEP surgery. Therefore, our results may not be applicable to younger patients, those undergoing other surgical methods (such as TAPP or open repair), or those treated with different mesh materials. Before these biomarkers can be applied in clinical practice, external validation through larger-scale, multicenter, and more diverse cohorts is necessary. Fourth, we did not assess preoperative pain status or psychological factors (such as anxiety, depression, and pain catastrophizing), which are known predictors of chronic postoperative pain (Feyizi et al., 2025; Ghoshal et al., 2023). These unmeasured variables may confound the observed correlations; thus, future research should include a comprehensive assessment of these factors to more accurately elucidate the specific role of inflammatory mediators in the development of chronic postoperative pain.

## 5 Conclusion

The incidence of chronic inguinal pain after laparoscopic TEP surgery in elderly patients is 9.5%. Postoperative serum IL-6 and NLRP3 levels within 24 h were identified as potential independent risk factors for chronic pain and demonstrated excellent predictive performance (AUCs of 0.975 and 0.907, respectively), making them potential clinical biomarkers. Future research should involve larger-sample multicenter studies to further validate these findings and explore the potential value of targeting the IL-6/NLRP3 inflammatory pathway in the prevention and treatment of chronic postoperative pain.

## Data Availability

The original contributions presented in the study are included in the article/Supplementary material, further inquiries can be directed to the corresponding author.

## References

[ref1] Al-Sharabi A. Xu Y. Al-Danakh A. Xu Z. H. Kang H. N. Wen G. Y. . (2025). Laparoscopic vs. open inguinal hernia repair outcomes in patients aged 65 years and older. Front. Surg. 12:1610358. doi: 10.3389/fsurg.2025.1610358, 41438695 PMC12719437

[ref2] Andresen K. Rosenberg J. (2024). Transabdominal pre-peritoneal (TAPP) versus totally extraperitoneal (TEP) laparoscopic techniques for inguinal hernia repair. Cochrane Database Syst. Rev. 7:CD004703. doi: 10.1002/14651858.CD004703.pub3, 38963034 PMC11223180

[ref3] Beel E. Berrevoet F. (2022). Surgical treatment for chronic pain after inguinal hernia repair: a systematic literature review. Langenbeck's Arch. Surg. 407, 541–548. doi: 10.1007/s00423-021-02311-9, 34471953

[ref4] Cancer Genome Center (2024). Surgical technique and chronic postoperative inguinal pain in patients undergoing open inguinal hernioplasty in Portugal: a prospective multicentric cohort study. Acta Medica Port. 37, 507–517. doi: 10.20344/amp.20277, 38950617

[ref5] Chen C. Smith M. T. (2023). The NLRP3 inflammasome: role in the pathobiology of chronic pain. Inflammopharmacology 31, 1589–1603. doi: 10.1007/s10787-023-01235-8, 37106238 PMC10352419

[ref6] Chu X. Zhao R. Wang T. Chen X. Kang H. (2026). Peripheral inflammation and central sensitization associated with postoperative pain following arthroscopy surgery in rotator cuff injury. Radiol Med. 131, 147–157. doi: 10.1007/s11547-025-02079-8, 41045353

[ref7] Chu Z. Zheng B. Yan L. (2024). Incidence and predictors of chronic pain after inguinal hernia surgery: a systematic review and meta-analysis. Hernia J Hernias Abdom Wall Surg. 28, 967–987. doi: 10.1007/s10029-024-02980-7, 38538812

[ref8] de Goede B. Timmermans L. van Kempen B. J. H. van Rooij F. J. A. Kazemier G. Lange J. F. . (2015). Risk factors for inguinal hernia in middle-aged and elderly men: results from the Rotterdam study. Surgery 157, 540–546. doi: 10.1016/j.surg.2014.09.029, 25596770

[ref9] Dubský M. Fejfarova V. Bem R. Jude E. B. (2022). Pain Management in Older Adults with chronic wounds. Drugs Aging 39, 619–629. doi: 10.1007/s40266-022-00963-w, 35829959

[ref10] Feyizi H. Bilen A. Karaoğlu R. O. (2025). Impact of preoperative anxiety on chronic postoperative pain after inguinal hernia repair. Hernia 29:310. doi: 10.1007/s10029-025-03502-9 41123683

[ref11] Forester B. Attaar M. Lach M. Chirayil S. Kuchta K. Denham W. . (2022). Inguinal hernia mesh is safe in 1720 patients. Surg. Endosc. 36, 1609–1618. doi: 10.1007/s00464-021-08442-w, 33763744

[ref12] Ghoshal A. Bhanvadia S. Singh S. Yaeger L. Haroutounian S. (2023). Factors associated with persistent postsurgical pain after total knee or hip joint replacement: a systematic review and meta-analysis. Pain Rep. 8:e1052. doi: 10.1097/PR9.0000000000001052, 36699992 PMC9833456

[ref13] Gianetta E. de Cian F. Cuneo S. Friedman D. Vitale B. Marinari G. . (1997). Hernia repair in elderly patients. Br. J. Surg. 84, 983–985. doi: 10.1002/bjs.1800840721, 9240142

[ref14] Halpern A. I. Klein M. McSweeney B. Tran H. V. Ganguli S. Haney V. . (2024). Trends in minimally invasive and open inguinal hernia repair: an analysis of ACGME general surgery case logs. Surg. Endosc. 38, 2344–2349. doi: 10.1007/s00464-024-10805-y, 38632119

[ref15] Haque M. R. Rahman M. S. Hossain M. S. Khan L. N. (2022). Comparison between Lichtenstein and laparoscopic totally extraperitonial (TEP) tension free mesh repair of inguinal hernia. Mymensingh Med J MMJ. 31, 1128–1134. 36189562

[ref16] Heniford B. T. Walters A. L. Lincourt A. E. Novitsky Y. W. Hope W. W. Kercher K. W. (2008). Comparison of generic versus specific quality-of-life scales for mesh hernia repairs. J. Am. Coll. Surg. 206, 638–644. doi: 10.1016/j.jamcollsurg.2007.11.025, 18387468

[ref17] Hernandez-Rosa J. Lo C. C. Choi J. J. Colon M. J. Boudourakis L. Telem D. A. . (2011). Laparoscopic versus open inguinal hernia repair in octogenarians. Hernia 15, 655–658. doi: 10.1007/s10029-011-0838-5, 21691736

[ref18] Hu T. Sun Q. Gou Y. Zhang Y. Ding Y. Ma Y. . (2022). Salidroside alleviates chronic constriction injury-induced neuropathic pain and inhibits of TXNIP/NLRP3 pathway. Neurochem. Res. 47, 493–502. doi: 10.1007/s11064-021-03459-y, 34626306

[ref19] Karatas T. Selcuk E. B. Karatas M. Tahtali I. N. Yildirim A. Arslan A. K. (2023). Evaluation of risk factors for necrotic tissue resections in elderly patients with groin hernia. Ann. Ital. Chir. 94, 472–477. 37199472

[ref20] Kui W. Li Y. Gu Z. Xie L. Huang A. Kong S. . (2025). Electroacupuncture inhibits NLRP3-mediated microglial Pyroptosis to ameliorate chronic neuropathic pain in rats. J. Pain Res. 18, 1115–1129. doi: 10.2147/JPR.S506569, 40070891 PMC11895692

[ref21] Lee J. Y. Park C. S. Seo K. J. Kim I. Y. Han S. Youn I. . (2023). IL-6/JAK2/STAT3 axis mediates neuropathic pain by regulating astrocyte and microglia activation after spinal cord injury. Exp. Neurol. 370:114576. doi: 10.1016/j.expneurol.2023.114576, 37863306

[ref22] Liew W. Wai Y. Y. Kosai N. R. Gendeh H. S. (2017). Tackers versus glue mesh fixation: an objective assessment of postoperative acute and chronic pain using inflammatory markers. Hernia J Hernias Abdom Wall Surg. 21, 549–554. doi: 10.1007/s10029-017-1611-1, 28417279

[ref23] Loos M. J. A. Roumen R. M. H. Scheltinga M. R. M. (2007). Classifying Postherniorrhaphy pain syndromes following elective inguinal hernia repair. World J. Surg. 31, 1760–1765. doi: 10.1007/s00268-007-9121-4, 17566818

[ref24] Luo X. Tai W. L. Sun L. Pan Z. Xia Z. Chung S. K. . (2016). Crosstalk between astrocytic CXCL12 and microglial CXCR4 contributes to the development of neuropathic pain. Mol. Pain 12:1744806916636385. doi: 10.1177/1744806916636385, 27030717 PMC4956184

[ref25] Manangi M. Shivashankar S. Vijayakumar A. (2014). Chronic pain after inguinal hernia repair. Int. Sch. Res. Not. 2014:839681. doi: 10.1155/2014/839681 PMC4897331 27437477

[ref26] McMasters M. Mora J. (2025). Addressing meta-inflammation in the comprehensive management of chronic pain. Cureus 17:e94863. doi: 10.7759/cureus.94863, 41257114 PMC12621917

[ref27] Meng Y. Zhuang L. Xue Q. Zhang J. Yu B. (2021). NLRP3-mediated neuroinflammation exacerbates incisional hyperalgesia and prolongs recovery after surgery in chronic stressed rats. Pain Physician 24, E1099–E1108. 34704719

[ref28] Mokhtari T. Irandoost E. Sheikhbahaei F. (2024). Stress, pain, anxiety, and depression in endometriosis-targeting glial activation and inflammation. Int. Immunopharmacol. 132:111942. doi: 10.1016/j.intimp.2024.111942, 38565045

[ref29] Patel L. Y. Lapin B. Gitelis M. E. Brown C. Linn J. G. Haggerty S. . (2017). Long-term patterns and predictors of pain following laparoscopic inguinal hernia repair: a patient-centered analysis. Surg. Endosc. 31, 2109–2121. doi: 10.1007/s00464-016-5207-0, 27585467

[ref30] Sonowal H. Zhang H. Rice W. Howell S. B. S H. Z H. . (2022). Luxeptinib disables NLRP3 inflammasome-mediated IL-1β release and pathways required for secretion of inflammatory cytokines IL-6 and TNFα. Biochem. Pharmacol. 195:114861. doi: 10.1016/j.bcp.2021.114861 34843717

[ref31] Sanders D. L. Kingsnorth A. N. (2012). The modern management of incisional hernias. BMJ 344:e2843. doi: 10.1136/bmj.e2843, 22573647

[ref32] Ulutas M. E. Yilmaz A. H. (2025). Comparison of open and laparoscopic inguinal hernia repair in the elderly patients: a randomized controlled trial. Hernia 29:179. doi: 10.1007/s10029-025-03368-x, 40407912 PMC12101994

[ref33] Wang F. Ma B. Ma Q. Liu X. (2030). Global, regional, and national burden of inguinal, femoral, and abdominal hernias: a systematic analysis of prevalence, incidence, deaths, and DALYs with projections to 2030. Int. J. Surg. 110, 1951–1967. doi: 10.1097/JS9.0000000000001071, 38265437 PMC11020045

[ref34] Wang H. Wang Z. Wang L. Sun L. Liu W. Li Q. . (2020). IL-6 promotes collagen-induced arthritis by activating the NLRP3 inflammasome through the cathepsin B/S100A9-mediated pathway. Int. Immunopharmacol. 88:106985. doi: 10.1016/j.intimp.2020.106985, 33182050

[ref35] Wang Y. Zhou X. Zhou X. (2024). Correlation analysis between the early diagnosis and severity of neuropathic pain and the activation level of NLRP3 inflammasome: a retrospective study. Medicine 103:e38356. doi: 10.1097/MD.0000000000038356, 38996109 PMC11245240

[ref36] Widder A. Reese L. Lock J. F. Wiegering A. Germer C. T. Kindl G. K. . (2023). Postoperative analgesics score as a predictor of chronic postoperative inguinal pain after inguinal hernia repair: lessons learned from a retrospective analysis. World J. Surg. 47, 2436–2443. doi: 10.1007/s00268-023-07074-6, 37248322 PMC10474177

[ref37] Wu J. J. Baldwin B. C. Goldwater E. Counihan T. C. (2017). Should we perform elective inguinal hernia repair in the elderly? Hernia J Hernias Abdom Wall Surg. 21, 51–57. doi: 10.1007/s10029-016-1517-3, 27438793

[ref38] Zhang Y. Chen R. Hu Q. Wang J. Nie H. Yin C. . (2022). Electroacupuncture ameliorates mechanical allodynia of a rat model of CRPS-I via suppressing NLRP3 inflammasome activation in spinal cord dorsal horn neurons. Front. Cell. Neurosci. 16:826777. doi: 10.3389/fncel.2022.826777, 35693886 PMC9174662

[ref39] Zhong S. Sun Q. Wen J. Zhang Z. Chen Y. Ye H. . (2024). Dexmedetomidine attenuates inflammatory response and chronic pain following video-assisted thoracoscopic surgery for lung cancer. Surgery 176, 1263–1272. doi: 10.1016/j.surg.2024.06.001, 38997865

