# Supplementary material for: Associations between serum inflammatory mediators and chronic pain after laparoscopic inguinal hernia repair in elderly patients
Source: Front Hum Neurosci. 2026 Aug 3;20:1846116. doi: 10.3389/fnhum.2026.1846116 (PMC13479288; doi:10.3389/fnhum.2026.1846116)
Supplement: Supplementary file 1 [file Table_1.docx]

**Supplementary Table 1. Bootstrap internal validation and model calibration**

| Model | Predictors | Original AUC | Optimism-corrected AUC | HL χ² | HL df | HL P value |
| --- | --- | --- | --- | --- | --- | --- |
| Model 1 | Hernia size + IL-6 | 0.975 | 0.959 | 0.74 | 6 | 0.994 |
| Model 2 | Hernia size + NLRP3 | 0.907 | 0.89 | 4.285 | 6 | 0.638 |

Bootstrap internal validation was performed with 1,000 resampling iterations. The optimism-corrected AUC was calculated as follows: 2 × original AUC − bootstrap mean AUC. A Hosmer–Lemeshow (HL) test result of P > 0.05 indicates good model calibration. AUC, area under the curve. This table is presented as supplementary material.
